# Heartfelt empathy? No association between interoceptive awareness, questionnaire measures of empathy, reading the mind in the eyes task or the director task

**DOI:** 10.3389/fpsyg.2015.00554

**Published:** 2015-05-01

**Authors:** Vivien Ainley, Lara Maister, Manos Tsakiris

**Affiliations:** Lab of Action and Body, Department of Psychology, Royal Holloway University of London, EghamUK

**Keywords:** empathy, perspective-taking, emotion recognition, interoception, heartbeat perception

## Abstract

Interoception, defined as afferent information arising from within the body, is the basis of all emotional experience and underpins the ‘self.’ However, people vary in the extent to which interoceptive signals reach awareness. This trait modulates both their experience of emotion and their ability to distinguish ‘self’ from ‘other’ in multisensory contexts. The experience of emotion and the degree of self/other distinction or overlap are similarly fundamental to empathy, which is an umbrella term comprising affect sharing, empathic concern and perspective-taking (PT). A link has therefore often been assumed between interoceptive awareness (IA) and empathy despite a lack of clear evidence. To test the hypothesis that individual differences in both traits should correlate, we measured IA in four experiments, using a well-validated heartbeat perception task, and compared this with scores on several tests that relate to various aspects of empathy. We firstly measured scores on the Index of Interpersonal Reactivity and secondly on the Questionnaire of Cognitive and Affective Empathy. Thirdly, because the ‘simulationist’ account assumes that affect sharing is involved in recognizing emotion, we employed the ‘Reading the Mind in the Eyes Task’ for the recognition of facial expressions. Contrary to expectation, we found no significant relationships between IA and any aspect of these measures. This striking lack of direct links has important consequences for hypotheses about the extent to which empathy is necessarily embodied. Finally, to assess cognitive PT ability, which specifically requires self/other distinction, we used the ‘Director Task’ but found no relationship. We conclude that the abilities that make up empathy are potentially related to IA in a variety of conflicting ways, such that a direct association between IA and various components of empathy has yet to be established.

## Introduction

Empathy is an essential aspect of human emotional experience and social interaction (Panksepp, 1998; Preston and de Waal, 2002; Gonzalez-Liencres et al., 2013). It is, however, a notoriously difficult concept to define and operationalise (Singer and Lamm, 2009; Decety and Cowell, 2014). We adopt in this paper a definition of empathy as an umbrella term comprising ‘affect sharing,’ otherwise known as ‘Emotion Contagion’ [which can lead to ‘personal distress (PD)’], ‘empathic concern (EC)’ (defined as the motivation to care for others) and ‘perspective-taking (PT),’ which is putting oneself in the other’s emotional shoes (Bernhardt and Singer, 2012; Decety and Cowell, 2014).

Of these three components, affect sharing is assumed to be an automatic process, whereby perceiving or imagining another person in a particular emotional state activates the same state in the observer, producing similar autonomic and somatic responses (Preston and Hofelich, 2012), such that the emphasiser ‘feels with’ the person in distress (Singer and Lamm, 2009).

‘Empathic concern,’ which is also called ‘sympathy’ or ‘compassion,’ involves ‘feeling for’ the other person (Singer and Lamm, 2009) and is associated with motivation to alleviate their suffering (Singer and Lamm, 2009; Decety and Cowell, 2014). This construct is frequently equated with empathy, although it does not necessarily involve any affect sharing (Bernhardt and Singer, 2012).

During ‘PT’ the observer consciously puts herself in the shoes of the person observed (Jackson et al., 2006; Lamm et al., 2007). This is closely related to theory of mind – the distinction being that during the PT involved in empathy the observer must understand the feelings of another person, whereas theory of mind is defined as understanding the other’s beliefs (Lawrence et al., 2004; Preston and Hofelich, 2012).

All three components of empathy involve a process whereby the empathiser represents both ‘self’ and ‘other’ (the person observed). This process may be regarded as a continuum. Overlapping representations of self and other are involved in affect sharing. By contrast, accurate self/other distinction (between one’s own and the other’s viewpoint) is required for PT (Bird et al., 2010). It is unclear how self/other overlap or self/other distinction impacts on EC. On the one hand EC may be a function of previous affect sharing and therefore involve self/other overlap. However, it might equally depend on good self/other distinction if, for example, affect sharing leads to PD which may interfere with the motivation to help (Singer and Klimecki, 2013).

A link between empathy and ‘interoceptive awareness (IA)’ can be predicted, on the basis of both affect sharing and self/other distinction (or overlap), where IA is defined as the extent to which internal bodily cues reach awareness and influence behavior, feelings and cognition. An extensive literature shows that IA influences the intensity of an individual’s own emotional experience (Barrett et al., 2004; Pollatos et al., 2005; Wiens, 2005). For example, people with high IA report more emotional arousal for identical changes in objective physiological indices (Wiens, 2005; Pollatos and Schandry, 2008; Dunn et al., 2010). This implies that high trait IA could be associated with greater affect sharing, because the shared emotion is potentially more intense than it would be in an individual with low IA. Moreover, self/other distinction (or overlap), which is also crucial to empathy, is modulated by IA, specifically in embodied contexts. Individuals with good IA are less susceptible to body ownership illusions (Tsakiris et al., 2011; Tajadura-Jiménez and Tsakiris, 2013) and when interoceptive cues are projected onto a virtual image of their own body, or hand, people have a greater sense of self-identification with, and self-location toward, that image (Aspell et al., 2013; Suzuki et al., 2013). This suggests that IA is associated with better self/other distinction and will therefore correlate positively with PT, although a relationship with EC cannot be unambiguously predicted.

The small number of studies that have attempted to link interoception with empathy provide inconclusive evidence because of differences in the way that both empathy and IA have been operationalised and measured. For example, Fukushima et al. (2011) reported that the amplitude of heartbeat-evoked potentials (HEPs; a potential index of interoceptive cortical processing) was higher when participants judged emotion in a pictured face, compared with judgments of facial symmetry. Ernst et al. (2013) found that when participants were asked to empathize with emotional facial expressions after a period of explicit interoceptive attention, neural activity was enhanced in a number of brain regions involved in interoception and self-processing, compared with a control task of counting exteroceptive tones. These two experiments, however, used indirect tests of *both* IA and empathy. Other studies that compared the two traits using conventional heartbeat perception measures of IA reported conflicting results. Tajadura-Jiménez and Tsakiris (2013) reported correlations between IA and both the PT and Fantasy (FS) subscales of the Interpersonal Reactivity Index (IRI; Davis, 1983). Both of these measures suggest better self/other distinction in people with high IA but the relationships were significant only at the 10% level (Tajadura-Jiménez and Tsakiris, 2013). More recently, Grynberg and Pollatos (2015) found that participants with good IA, who viewed pictures of people in painful situations, rated the pain as greater and felt more compassion for the sufferer, although they reported no greater PD than controls. This result is potentially attributable to the greater emotional arousal also reported by the participants with high IA, which is typical of good heartbeat perceivers and could reflect affect sharing.

Given these conflicting results, we devised a set of experiments in an attempt to elucidate various strands of the potential relationship between IA and the components of empathy, measuring the former by the Mental Tracking Method of heartbeat perception (Schandry, 1981). Empathy has been assessed in many different ways. The measures chosen in the four studies described in this paper were selected as widely used measures of affect sharing, EC and PT and for being relevant to previous empirical research purporting to show links between interoception and empathy.

We first chose the most prominent self-report empathy scale, which is the IRI (Davis, 1983). Its subscales include EC, PT and PD, all of which refer to aspects of empathy that have been described in theoretical accounts. The EC subscale of the IRI has been particularly widely used as a general empathy measure (for a review see Bernhardt and Singer, 2012). The IRI, however, possess no specific measure of affect sharing. We therefore selected as our second measure the recently developed Questionnaire of Cognitive and Affective Empathy (QCAE; Reniers et al., 2011) which includes a subscale for ‘Emotion Contagion’ (affect sharing) and has the additional merit of effectively summarizing several common self-report empathy measures.

Our third measure was also designed to assess affect sharing, reflecting the proposition that people understand and interpret emotional facial expressions by automatically simulating (i.e., by empathizing with) the observed expression (Preston and de Waal, 2002; Kaplan and Iacoboni, 2006). Although this ‘simulation account’ has been disputed (Gallese and Sinigaglia, 2011), it implies that people with high IA, who experience their own emotions more strongly, are likely to perform well when recognizing facial expressions in the ‘Reading the Mind in the Eyes’ test (Baron-Cohen et al., 2001). A further reason for choosing this measure was that the two prominent studies, described above, that report significant relationships between empathy and interoception have used similar facial stimuli (Fukushima et al., 2011; Ernst et al., 2013). Furthermore, it has been reported that people with high IA have an advantage in recognizing the presence of emotion in faces, which might imply that they are sharing affect by mirroring the observed emotion (Terasawa et al., 2014).

Finally, as a direct test of whether people with high IA are better at separating self from other in domains other than multisensory integration, we employed the ‘Director Task,’ which measures cognitive PT ability (Santiesteban et al., 2012).

The four experiments were approved by the Ethics Committee of the psychology department at Royal Holloway University of London. All participants gave written informed consent and were free to withdraw at will.

## Experiment 1

### Method

#### Participants

Ninety students at Royal Holloway University of London took part for course credit. The data for four participants was excluded because movement artifacts made it impossible to count the number of recorded heartbeats. Of the remaining 86 (14 men), mean age was 20.4 years (SD = 6.5).

#### Procedure

Participants completed the IRI after the heartbeat perception task.

#### Interoceptive Awareness (IA)

Interoceptive awareness was measured using the Mental Tracking Method of heartbeat perception (Schandry, 1981). Heartbeat perception methods correlate with awareness of gastric cues (Whitehead and Drescher, 1980; Herbert et al., 2012). The Mental Tracking Method has been extensively used in research on emotion (Dunn et al., 2007; Herbert et al., 2010; Werner et al., 2010; Pollatos et al., 2012). It is well-validated, with good test–retest reliability and discriminates well between individuals (Mussgay et al., 1999; Werner et al., 2013). Throughout this paper ‘IA’ refers to the accuracy with which participants were able to count their own heartbeats on the Mental Tracking task (Cuenen et al., 2012; Garfinkel and Critchley, 2013).

Participants were seated in a comfortable chair and given several minutes to relax, in preparation for the task. All instructions were delivered and behavioral responses were recorded using Presentation software (Neurobehavioral Systems, Albany, CA, USA) on a standard desktop PC. Instructions were presented over noise-attenuating headphones. The onset and offset of each heartbeat counting trial were cued by the words ‘*go’* and *‘stop,’* presented audiovisually. Results are sensitive to the instructions given (Ehlers et al., 1995) so a standard instruction was used, whereby participants were asked to concentrate hard and try to silently count their own heartbeats, simply by *‘listening’* to their bodies, without taking their pulse. Heartbeat signals were acquired with a piezo-electric pulse transducer, fitted to the participant’s left index finger and connected to a physiological data unit (26T PowerLab, AD Instruments), sampling at 1 kHz, which recorded the derived electrical signal onto a second PC running LabChart6 software (AD Instruments). Three trials (25, 35, 45 s) were presented in random order, after one training interval of 15 s. No feedback was given. IA was calculated as [1/3Σ (1-(|recorded heartbeats – counted heartbeats| /recorded heartbeats); Schandry, 1981)]. Higher scores indicate higher IA. Heart rate is known to correlate with IA and this was also recorded during the heartbeat perception task (Cameron, 2001; Knapp-Kline and Kline, 2005; Ainley et al., 2012).

#### The Interpersonal Reactivity Index

The IRI (Davis, 1983) is a prominent, well-validated and reliable measure of trait empathy, containing four 7-item subscales. These are EC, PT, PD, and Fantasy. Typical items are: for EC *‘I often have tender, concerned feelings for people less fortunate than me’*; for PT *‘I believe that there are two sides to every question and try to look at them both’*; for PD *‘When I see someone who badly needs help in an emergency, I go to pieces’*; and for Fantasy *‘I really get involved with the feelings of the characters in a novel.’* Each item is scored on a five-point Likert scale from 0 for ‘*does not describe me well’* to 4 *‘describes me very well.’*

### Results

The mean values and SD obtained were close to published norms (**Table 1**).

**Table 1:** Descriptive statistics for the Interpersonal Reactivity Index (IRI) and its subscales – empathic concern (EC), personal distress (PD), perspective-taking (PT) and fantasy (FS).

	IRI	EC	PD	PT	FS
Mean (SD)	65.8 (15.0)	19.9 (4.6)	11.7 (5.6)	17.2 (4.9)	16.8 (4.9)
Norms (a)		20.36 (4.02)	10.87 (4.78)	17.37 (4.79)	

*(a) Norms value provided by Davis as personal communication, reported in Belllini et al. (2002)*.

Correlations between IA and the IRI scores are shown in **Table 2**. Bonferroni corrections for multiple comparisons were applied with an alpha value of 0.01. There were no significant correlations.

**Table 2:** Correlations between interoceptive awareness (IA), the Interpersonal Reactivity Index (IRI) and its subscales.

	IRI	EC	PD	PT	FS
Correlations with IA (all participants) *(n = 86)*	*r* = -0.10, *p* = 0.36	*r* = 0.002, *p* = 0.98	*r* = -0.12, *p* = 0.28	*r* = 0.10, *p* = 0.34	*r* = -0.23, *p* = 0.03^*^
Correlations with IA (women only) *(n = 72)*	*r* = -0.14, *p* = 0.23	*r* = 0.04, *p* = 0.75	*r* = -0.08, *p* = 0.50	*r* = 0.05, *p* = 0.66	*r* = -0.27, *p* = 0.02^*^

^*^non-significant after Bonferroni correction.

Mean IA was 0.64 (SD = 0.18). IA was negatively correlated with the participants’ heart rate, *r* = -0.47, *p* < 0.001. Heart rate was not significantly correlated with any IRI measure.

In this sample there were significant differences in mean IRI scores between genders, *F*(1,84) = 11.7, *p* = 0.001. However, the small number of men amongst the participants makes this comparison unreliable. Correlations for the 72 women participants only were therefore also calculated. An apparent negative correlation between IA and Fantasy did not survive Bonferroni correction, with an alpha level of 0.01. No other correlation was significant.

### Discussion of Experiment 1

We investigated the relationship between a standard measure of IA and the most commonly used self-report measure of empathy. There were no significant correlations between IA and the IRI as a whole or with any of its subscales. The only relationship that approached significance was a negative correlation with Fantasy, suggesting that people with high IA tend not put themselves in the position of characters in books and films, which might perhaps reflect good self/other distinction. However, this did not survive Bonferroni correction.

Although the IRI has the advantage of distinct, separable subscales that refer to several theoretically based components of empathy, direct mapping of each subscale onto a particular aspect of empathy has been criticized (Singer and Lamm, 2009). The EC score is a very widely used measure of empathy but did not correlate with IA in this study. In order to verify the null results found in Experiment 1, we selected a further self-report measure – the QCAE (Reniers et al., 2011), which, unlike the IRI, has a specific subscale designed to capture affect sharing.

## Experiment 2

### Method

#### Participants

Thirty-four students (five men), mean age 20.2 years (SD = 2.9), at Royal Holloway University of London, took part for payment. There were no exclusions.

#### Procedure

Interoceptive awareness was measured as in Experiment 1. Participants completed the QCAE after the heartbeat perception task.

#### Questionnaire of Cognitive and Affective Empathy

The QCAE (Reniers et al., 2011) is an attempt to capitalize on the strengths of several commonly used self-report measures. It is comprised of items from the Empathy Quotient (Lawrence et al., 2004), the Hogan Empathy Scale (Hogan, 1969), the Empathy subscale of the Impulsiveness-Venturesomeness-Empathy Inventory (Esysenck and Eysenck, 1978), and the IRI (Davis, 1983). The scale consists of 31 items to which participants respond on a four-point Likert scale from *‘strongly disagree’* to *‘strongly agree.’* ‘Cognitive Empathy’ is made up of two subscales which are (i) ‘PT’ (defined as ‘Intuitively putting oneself in another person’s shoes’), assessed by 10 items, e.g., *‘I can easily work out what another person might want to talk about’*; and (ii) ‘Online Simulation’ (defined as ‘An effortful attempt to put oneself in another person’s position by imagining what that person is feeling’), assessed by nine items, e.g., *‘I try to look at everyone’s side of a disagreement before I make a decision.’* By contrast, ‘Affective Empathy’ is made up of three subscales, which are (iii) ‘Emotion Contagion’ (defined as ‘The automatic mirroring of the feelings of others’), assessed by four items, e.g., *‘People I am with have a strong influence on my mood’*; (iv) ‘Proximal Responsivity’ (defined as ‘Affective responses when witnessing the mood of others in a close social context’), assessed by four items, e.g., *‘I am very unhappy when I see someone cry’*; and (v) Peripheral Responsivity (defined as ‘Similar to Proximal Responsivity but in a detached context’), assessed by four items, e.g., *‘I usually stay detached when watching a film.’*

### Results

The mean values for the QCAE scales were close to published norms (**Table 3**).

**Table 3:** Descriptive statistics for the Questionnaire of Cognitive and Affective Empathy (QCEA) and its subscales – cognitive empathy (Cog Emp) perspective-taking (PT), online simulation (Sim), affective empathy (Affect Emp), emotion contagion (Emot Con), proximal responsivity (Prox Res) and peripheral responsivity (Periph Res).

	QCAE	Cog Emp			Affect Emp			

			**PT**	**Sim**		**Emot Con**	**Prox Res**	**Periph Res**
Women Mean (SD)	95.6 (7.5)	59.6 (5.4)	31.9 (3.2)	27.7 (3.0)	35.9 (4.7)	11.9 (2.0)	12.5 (2.0)	11.6 (2.8)
Men Mean (SD)	84.6 (7.6)	55.4 (6.1)	28.8 (4.1)	26.6 (2.4)	29.2 (4.7)	9.0 (3.3)	10.2 (2.4)	10.0 (1.0)
Norms for Women (a)		59.4 (6.3)			36.7 (4.3)			
Norms for Men (a)		56.1 (10.5)			32.3 (6.3)			

*(a) Reported by Reniers et al. (2011) for University students, aged 20–30, *n* = 925*.

Correlations between IA and the QCAE scores are shown in **Table 4** for all participants and for the 29 women participants alone. IA was not significantly correlated with the whole QCAE scale nor with any of the subscale measures.

**Table 4:** Correlations between interoceptive awareness (IA) and the Questionnaire of Affective and Cognitive Empathy (QCAE) and its subscales.

	QCAE	Cog Emp			Affect Emp			

			**PT**	**Sim**		**Emot Con**	**Prox Resp**	**Periph Resp**
Correl. with IA *n* = 34	*r* = 0.09 *p* = 0.63	*r* = 0.07 *p* = 0.70	*r* = 0.18 *p* = 0.32	*r* = -0.08 *p* = 0.69	*r* = 0.06 *p* = 0.73	*r* = 0.13 *p* = 0.47	*r* = 0.01 *p* = 0.98	*r* < 0.01 *p* = 0.99
Correl. with IA (women) *n* = 29	*r* = -0.02 *p* = 0.91	*r* = -0.05 *p* = 0.79	*r* = 0.09 *p* = 0.64	*r* = -0.19 *p* = 0.32	*r* = 0.02 *p* = 0.91	*r* = 0.16 *p* = 0.42	*r* = -0.01 *p* = 0.96	*r* = -0.07 *p* = 0.73

Mean IA was 0.61 (SD = 0.16). IA was negatively correlated with average resting heart rate, for all participants, *r* = -0.37, *p* = 0.03, and for the women participants alone, *r* = -0.45, *p* = 0.02, as in Experiment 1. Correlations between heart rate and scores on the QCAE and its subscales were non-significant (Bonferroni corrections with an alpha value of 0.006 were applied).

### Discussion of Experiment 2

No links were found between IA and any scale of the QCAE. In particular, Emotion Contagion (which measure affect sharing) was not related to IA. The results of Experiment 2 thus confirm those of Experiment 1 and might be explained by the general weakness of self-report measures. Questionnaires can only reflect what people think they feel, rather than what they would truly feel in any given context. Such measures are open to the additional confound that they may index what is socially desirable, rather than recording the respondent’s true feelings (Singer and Lamm, 2009). The two further tests we used require judgments that avoid these two confounds. Given that several of the studies that have linked empathy and interoception have used tasks involving the appraisal of facial expressions which, it has been argued, involves affect sharing (Gallese and Sinigaglia, 2011), we next investigated whether scores on the ‘Reading the Mind in the Eyes’ test are modulated by IA.

## Experiment 3

### Method

#### Participants

One hundred and thirteen adult members of the public volunteered to participate in this study, as part of the Live Science installation at the Science Museum London. Sixteen participants were excluded before data analysis, due to incomplete data, movement artifacts or insufficient attention to the tasks, which left 97 participants (40 men) in the final data set. The mean age of these participants was 31.0 years (SD = 12.0).

#### Procedure

Interoceptive awareness was measured as in Experiment 1. Participants completed the Reading the Mind in the Eyes test after the heartbeat perception task.

#### ‘Reading the Mind in the Eyes’ Task

The Reading the Mind in the Eyes Task is a commonly used test that is the product of research into empathy deficits in autism. The procedure was developed by Baron-Cohen et al. (2001). Participants are seated at a standard PC and shown a series of 36 images of faces cropped around the eyes. For each image, participants are provided with four single words to describe the possible emotions that the eyes could be displaying (e.g., serious, ashamed, alarmed, or bewildered). They are required to choose which description best matches the emotion displayed in the image. The experiment is self-paced and takes about 7 min.

### Results

Interoceptive awareness was calculated as in Experiment 1. The mean IA score of this sample was 0.67 (SD = 0.17). As often reported, average heart rate was significantly negatively correlated with IA, *r* = -0.43, *p* < 0.001.

Performance on the Reading the Mind in the Eyes Task was assessed by calculating a simple accuracy score that indicated the proportion of the total trials in which the participant identified the correct emotion. Mean accuracy score was 0.74 (SD = 0.09). There were no gender differences in accuracy, *t*(95) = -0.36, *p* = 0.72. To identify links between IA and emotion recognition, a Pearson’s correlation coefficient was calculated between IA scores and Reading the Mind in the Eyes scores but this was non-significant, *r* = -0.10, *p* = 0.36.

### Discussion of Experiment 3

Although evidence suggests that individuals with high IA experience their own emotions as more intense than those with lower IA (Wiens et al., 2000), our findings replicate those of Hanford and colleagues who reported that this does not confer any benefits when people are required to recognize *others’* emotions in the Reading the Mind in the Eyes test (Handford et al., 2013). Their experiment, however, used a heartbeat discrimination task based on the Method of Constant Stimuli in which the majority of people score at chance (Lenggenhager et al., 2013; Eshkevari et al., 2014). We had expected that IA measured by the Mental Tracking Method (Schandry, 1981) might be a more successful index because this test produces a distribution of scores that is approximately Gaussian (Ainley et al., 2012). Terasawa’s task, using full faces and morphs containing variable amounts of emotion, also implies that there is a link between IA and recognition of emotion in faces but their results were significant only when comparing the performance of the 10 best and 10 worst heartbeat perceivers (Terasawa et al., 2014).

If the simulation account is correct in claiming that the identification of emotion in another person’s face relies on resonating with the observed emotion (Gallese, 2007; Gallese and Sinigaglia, 2011), then the findings of this experiment casts doubt on the hypothesis that interoception is linked to empathy. Alternatively the Reading the Mind in the Eyes test itself may be performed by theory of mind and therefore not involve the observer resonating with the observed emotion.

For our fourth and final experiment we tested IA against the ‘Director Task,’ which assesses cognitive PT (which requires self/other distinction). Research into bodily illusions has suggested that individuals with high IA may more clearly distinguish between their own and other’s perspectives in multisensory contexts (Tsakiris et al., 2011; Aspell et al., 2013). We wished to determine whether this advantage would translate to the Director Task, given that putting oneself in another’s *emotional* shoes is a facet of empathy.

## Experiment 4

### Method

#### Participants

Sixty-six adults volunteered for this study, as part of the Live Science installation at the Science Museum, London. Six participants were excluded before data analysis due to incomplete data and movement artifacts, which left 60 participants (34 men), mean age 30.8 years (SD = 13.3).

#### Procedure

Interoceptive awareness was measured as in Experiment 1. Participants completed the Director Task after the heartbeat perception task.

#### The ‘Director Task’

The Director Task requires participants to move objects on a computer screen according to verbal instructions from a ‘Director,’ delivered via headphones. Crucially, on critical trials the Director’s visual perspective on the objects that have to be moved differs from that of participant. Thus participants must understand the Director’s point of view in order to perform the task correctly.

The visual stimulus consisted of a 4 × 4 grid containing eight common objects. The image of a character, introduced as ‘the Director,’ appears behind the grid. On each trial, the Director would name one of the objects and instruct the participant to move it, using the computer mouse, either one space up, down, left, or right. Importantly, five of the spaces in the grid were occluded, so that the participant could see the objects situated in these spaces but the Director, who was standing behind the grid, could not. In experimental trials (‘E’ trials), there was a conflict between the participant’s and the Director’s perspective. An example of such a situation would be if the Director asked the participant to ‘move the small candle right,’ but the smallest candle that the participant could see was occluded from the Director. In this case, the participant should instead move the second smallest candle, which *can* be seen by the Director. In addition to these critical ‘E’ trials, there were also two types of control trial (‘C1’ and ‘C2’ trials). In these trials there was no conflict between the participant’s and Director’s perspectives. Thus participants were either asked to move an object which was the only item of that type and was visible to both participant and Director (C1 trials); or the participant was asked to move the smallest or largest of a type of object and again that target object was visible to both (C2 trials).

The procedure and stimuli were adapted from Santiesteban et al. (2012). For the participants in the current study, the task was shortened in make it more suitable for members the general public visiting the Science Museum. Participants completed 12 E trials, six C1 trials, and six C2 trials in total. The task took about 5 min. Participants’ accuracy and reaction times (RTs) were recorded.

### Results

Accuracy and RT data were analyzed with a repeated-measures ANOVA with trial type (E vs. C1 vs. C2) as the within-subjects factor. The data violated the sphericity assumption and therefore all results reported are with Greenhouse-Geisser corrections. For accuracy scores, there was a main effect of trial type, *F*(1.31,77.47) = 28.72, *p* < 0.001. *Post hoc* paired samples *t*-tests revealed that participants were significantly more accurate on C1 trials, *M* = 0.91, SD = 0.22, than they were on C2 trials, *M* = 0.61, SD = 0.17, *t*(59) = 12.19, *p* < 0.001, or on E trials, *M* = 0.61, SD = 0.44, *t*(59) = 5.39, *p* < 0.001. Performance did not differ significantly between C2 and E trials, *t*(59) = 0.02, *p* = 0.98. The same analysis was repeated with gender inserted as a between-groups factor. The main effect of trial type remained, and there was no effect of gender or any interaction.

For RT scores, there was also a main effect of trial type, *F*(1.31,95.76) = 40.03, *p* < 0.001. Paired samples *t*-tests revealed that responses on E trials, *M* = 4.54 s, SD = 1.20, were significantly slower than on both C1 trials, *M* = 3.79 s, SD = 1.04, *t*(59) = 7.30, *p*< 0.001, and on C2 trials, *M* = 3.75 s, SD = 0.88, *t*(59) = 6.74, *p* < 0.001. Performance on C1 and C2 trials did not differ significantly, *t*(59) = 0.38, *p* = 0.70. The same analysis was repeated with gender inserted as a between-groups factor. The main effect of trial type remained, and there was no main effect of gender or any significant interaction.

Interoceptive awareness was calculated as in Experiment 1. Mean IA was 0.71 (SD = 0.16). IA was negatively correlated with the participants’ heart rate, *r* = -0.33, *p* < 0.001. Heart rate was not significantly correlated with any aspect of performance on the Director Task.

To assess the relationship between performance on the Director Task and participants’ IA, we calculated Pearson’s correlation coefficients between participants’ performance on the E trials and their IA scores. We focussed on E trials, as it is only on these trials that the participant is required to take a perspective that conflicts with their own, which is the ability relevant to cognitive empathy.

No significant correlations were found between IA and performance on E trials, either as measured by RTs, *r* = -0.02, *p* = 0.91, or by accuracy, *r* = -0.08, *p* = 0.55. There was also no correlation between IA and performance on C1 or C2 trials (all *p*-values >0.47).

### Discussion of Experiment 4

Research into body ownership illusions has implied that people with high IA make better self/other distinctions in embodied contexts (Tsakiris et al., 2011; Aspell et al., 2013; Suzuki et al., 2013). Given that the ability to distinguish between one’s own and another’s perspective is crucial for successful performance on the Director Task (Santiesteban et al., 2012), we hypothesized that we would find a positive association between IA and performance on this task.

However, this association was not found. It is likely that the type of sensory and embodied self/other distinction modulated in bodily illusions does not carry over to the cognitive self/other distinction measured by the Director Task, which may be solved using theory of mind.

## General Discussion

We investigated the relationship between individual differences in IA and components of empathy, using a well-validated heartbeat perception task, together with four measures. In our first study we found no significant relationship with the IRI or any of its subscales, including EC and PT. In a further group of participants, no links were found between IA and scores on the QCAE or its subscales, including Emotion Contagion (which measures affect sharing) and PT. A further study found no significant relationship between IA and judgment of facial emotion in the Reading the Mind in the Eyes test (Baron-Cohen et al., 2001), which potentially depends on affect sharing, as a result of mirroring the observed emotion (Gallese and Sinigaglia, 2011). In our final experiment, scores on the Director Task which assesses cognitive PT (Santiesteban et al., 2012) were not associated with IA. Our only significant relationships were negative correlations between heart rate and IA, as frequently reported in other studies (Cameron, 2001; Knapp-Kline and Kline, 2005; Fairclough and Goodwin, 2007; Stevens et al., 2011), which potentially reflect physiological variables such as stroke volume of the heart.

These null findings imply that a simple relationship between IA and any of the three components of empathy cannot be predicted. The theoretical basis for supposed links rest on evidence that IA is related to the intensity of one’s own emotional experience. If empathy is embodied and involves the vicarious experience of emotion, then it was predicted that people with high IA would have a greater tendency to resonate with the emotion that they observe in others. They should hence demonstrate greater affect sharing and hence higher scores on the Emotion Contagion subscale of the QCAE as well as better ability in identifying emotional expressions in the Reading the Mind in the Eyes test, assuming that the task involves shared affect by mirroring of the observed emotion.

However, a previously overlooked issue, which may explain our results, is that it is by no means obvious how the better self/other distinction that is associated with high IA impacts on the components of empathy. If high IA is accompanied by less self/other overlap, this might involve *less* shared affect and consequently *less* experience of vicarious emotion. Thus if the effects of greater emotional arousal and greater self/other distinction work in opposite directions, as seems probable, then the predicted link between IA and affect sharing becomes a matter of which effect is stronger. We believe that this type of complexity explains our null results for the Emotion Contagion subscale of the QCAE and the Reading the Mind in the Eyes test. Support for his idea is provided by Grynberg and Pollatos’ (2015) study in which good heartbeat perceivers reported more compassion when viewing pictures of people in pain. Their results suggest that the higher arousal these participants also reported led them to judge the observed pain as more severe. However, they did not report greater PD, which may have been the result of good self/other distinction which allowed these individuals to avoid sharing the suffering. In support of this we found no relationship between IA and the PD subscale of the IRI.

Empathic concern (the motivation to help others) was not linked to IA in either questionnaire. EC is commonly used as a general measure of empathy. However, a link between the two would be anticipated only if EC is influenced by previous experience of affect sharing or, alternatively, by better self/other distinction (both of which are potentially greater in good heartbeat perceivers). Potentially, EC relies, at least in part, on theory of mind rather than direct affect sharing (Bernhardt and Singer, 2012), which would negate any obvious direct relationship with IA.

With regard to PT, we expected a relationship between IA and the *affective* PT subscales of the two questionnaires (which assess putting oneself in another’s emotional shoes), given that people with high IA make better self/other distinction on embodied contexts. That no relationship was found suggests that the type of PT measured by putting oneself in the other person’s emotional shoes may depend on theory of mind, rather than on an embodied response. By contrast, the Director Task is a good measure of *cognitive* PT (Decety and Cowell, 2014) but the test may also be solved by theory of mind (Santiesteban et al., 2014), in which case it too is not necessarily comparable with the type of embodied self/other distinction that is involved in body ownership.

The difference between the results presented here and experiments that have previously reported links between empathy and IA can be attributed to differences of definition and measurement. In Ernst et al.’s (2013) study, for example, a heartbeat-counting task primed emotion circuits in the insula such that the effects carry over into a subsequent emotion appraisal task. This significant finding shows that attention to interoception enhances subsequent emotion processing. However, the authors did not assess IA as generally defined (Garfinkel and Critchley, 2013) and did not establish whether IA, as a trait, is linked to empathy. A similar difficulty of definition arises with Fukushima et al.’s (2011) study, which likewise employed an emotional appraisal task. The amplitude of HEPs increased during this empathy manipulation. However, while the amplitude of the HEP is a useful index of interoceptive processing, it has not previously been taken as a measure of trait IA. Fukushima et al.’s (2011) experiment therefore also provides, at best, indirect evidence of a possible link between IA and empathy.

In addition to the criticism of our four measures that are addressed above, further limitations of our study are that we did not screen for personality variables, such as psychopathy, narcissism, altruism, agreeableness, or trait anxiety, which potentially moderate links between IA and empathy.

Our results support the characterisation of empathy as an umbrella term for a distributed set of mechanisms (Singer, 2006; Decety, 2010; Zahavi, 2012). Foremost of these is affect sharing, which may be linked to IA through greater intensity of emotional experience, although good self/other distinction potentially confounds the relationship. An important focus for future research is further direct tests of affect sharing, such as empathy for pain (Singer et al., 2004) and facial EMG (Lamm et al., 2008). EC, by contrast, is perhaps a product of affect sharing, self/other distinction and theory of mind, so that a clear relationship with IA cannot be predicted. Finally, PT is closely related to theory of mind and may not be influenced by the type of embodied self/other distinction that is modulated by IA in body ownership illusions. While it is therefore probable that people with high IA have the ability to use their bodies to simulate the emotions of other people, we conclude that they may do so only in certain contexts, so that reliable associations between empathy measures and IA cannot be predicted.

## Conflict of Interest Statement

The authors declare that the research was conducted in the absence of any commercial or financial relationships that could be construed as a potential conflict of interest.
